# An EWAS of dementia biomarkers and their associations with age, African ancestry, and PTSD

**DOI:** 10.1186/s13148-024-01649-3

**Published:** 2024-03-02

**Authors:** Mark W. Miller, Erika J. Wolf, Xiang Zhao, Mark W. Logue, Sage E. Hawn

**Affiliations:** 1 National Center for PTSD, VA Boston Healthcare System (116B-2), 150 S. Huntington Avenue, Boston, MA 02130 USA; 2 Department of Psychiatry, Boston University Chobanian and Avedisian School of Medicine, Boston, MA 02118 USA; 3 Biomedical Genetics, Boston University Chobanian and Avedisian School of Medicine, Boston, MA 02118 USA; 4 Department of Biostatistics, Boston University School of Public Health, Boston, MA 02118 USA; 5 Department of Psychology, Old Dominion University, Norfolk, VA 23529 USA

**Keywords:** Aβ40, Aβ42, GFAP, NfL, pTau-181, PTSD, EWAS, African ancestry, Plasma, Biomarkers, Dementia, Alzheimer’s disease, Structural equation model

## Abstract

**Background:**

Large-scale cohort and epidemiological studies suggest that PTSD confers risk for dementia in later life but the biological mechanisms underlying this association remain unknown. This study examined this question by assessing the influences of PTSD, *APOE* ε4 genotypes, DNA methylation, and other variables on the age- and dementia-associated biomarkers Aβ40, Aβ42, GFAP, NfL, and pTau-181 measured in plasma. Our primary hypothesis was that PTSD would be associated with elevated levels of these markers.

**Methods:**

Analyses were based on data from a PTSD-enriched cohort of 849 individuals. We began by performing factor analyses of the biomarkers, the results of which identified a two-factor solution. Drawing from the ATN research framework, we termed the first factor, defined by Aβ40 and Aβ42, “Factor A” and the second factor, defined by GFAP, NfL and pTau-181, “Factor TN.” Next, we performed epigenome-wide association analyses (EWAS) of the two-factor scores. Finally, using structural equation modeling (SEM), we evaluated (a) the influence of PTSD, age, *APOE* ε4 genotype and other covariates on levels of the ATN factors, and (b) tested the mediating influence of the EWAS-significant DNAm loci on these associations.

**Results:**

The Factor A EWAS identified one significant locus, *cg13053408*, in *FANCD2OS*. The Factor TN analysis identified 3 EWAS-significant associations: *cg26033520* near *ASCC1, cg23156469* in *FAM20B,* and *cg15356923* in *FAM19A4*. The SEM showed age to be related to both factors, more so with Factor TN (*β* = 0.581, *p* < 0.001) than Factor A (*β* = 0.330, *p* < 0.001). Genotype-determined African ancestry was associated with lower Factor A (*β* = 0.196, *p* < 0.001). Contrary to our primary hypothesis, we found a modest negative bivariate correlation between PTSD and the TN factor scores (*r* = − 0.133, *p* < 0.001) attributable primarily to reduced levels of GFAP (*r* = − 0.128, *p* < 0.001).

**Conclusions:**

This study identified novel epigenetic associations with ATN biomarkers and demonstrated robust age and ancestral associations that will be essential to consider in future efforts to develop the clinical applications of these tests. The association between PTSD and reduced GFAP, which has been reported previously, warrants further investigation.

**Supplementary Information:**

The online version contains supplementary material available at 10.1186/s13148-024-01649-3.

## Background

Large-scale cohort and epidemiological studies suggest that stressful life events [1], psychological distress [2], and PTSD [3–6] confer increased risk for Alzheimer’s disease (AD) and related dementias (ADRD) in later life. However, the causal mechanisms underlying this association, including the influence of genetic and epigenetic factors on the relationship between PTSD and ADRD, remain an open question. This study was designed to address this by examining age- and AD-associated biomarkers in an age- and ancestrally-diverse cohort with a high prevalence of trauma exposure and PTSD.

In the past decade, considerable progress has been made in the identification of ADRD biomarkers. The neuropathological features of AD include the presence of amyloid β plaques and neurofibrillary tangles containing hyperphosphorylated tau. Amyloid positron emission tomography (PET) has been shown to offer a valid in vivo tool for assessing the presence of amyloid β deposits, and levels of the amyloid-beta peptide Aβ42 (or the Aβ42/Aβ40 ratio) in cerebrospinal fluid (CSF) also provide a valid indicator of the pathologic state of cerebral Aβ [7]. However, the high cost and invasiveness of PET and CSF assessment have motivated the search for inexpensive, minimally invasive, and objective peripheral biomarkers of ADRD that can be used not only to aid in diagnosis, but also for prognostic evaluation, tracking treatment response, and monitoring of disease progression.

The development of ultra-sensitive immunoassay technologies, such as the single-molecule digital assay (Simoa) by the Quanterix Corporation, now permits the reliable assessment of central nervous system (CNS)-derived proteins in blood samples at extremely low concentrations. The Simoa markers most relevant to ADRD, many not previously detectable in blood due to their low concentrations (or even known to exist in the periphery), include Aβ40 and Aβ42, glial fibrillary acidic protein (GFAP), neurofilament light chain (NfL), and phosphorylated tau at threonine 181 (pTau^181^). Aβ peptides are the main components of senile plaques and cleaved from the amyloid precursor protein into 40 and 42 amino acid residual peptides termed Aβ40 and Aβ42, respectively. Lower plasma Aβ42 and a lower Aβ42/Aβ40 ratio is associated with greater brain amyloid pathology and thought to be attributable to the aggregation of Aβ42 into amyloid plaques [8]. GFAP is a protein expressed in astrocytes and released during astrocytic activation. Elevated GFAP levels have been observed in association with traumatic brain injury and several neuroinflammatory and neurodegenerative CNS diseases [9]. NfL is a cylindrical protein that provides structural stability to, and enables the growth of, myelinated axons. It has been shown to provide a reliable index of axonal injury across several neurological disorders [10]. Finally, pTau^181^ is a form of phosphorylated tau that aggregates into deposits that form the neurofibrillary tangles characteristic of Alzheimer’s Disease and other tauopathies [11]. Each of these biomarkers has also been shown to increase as a function of age [12–14].

On the basis of prior research suggesting that PTSD is associated with risk for dementia, accelerated DNAm age, and other indices of advanced cellular aging [15–17], we hypothesized that aging patients with PTSD would show elevated levels of some or all of the brain age- and dementia-associated plasma biomarkers Aβ40, Aβ42, GFAP, NfL, and pTau^181^. Further, in light of recent evidence that the risk that PTSD confers for ADRD increases additively as a function the apolipoprotein E gene ε4 risk allele (*APOE* ε4) [3], we also evaluated the main and interactive effects of this important genetic risk factor. Specifically, we hypothesized that the strength of the association between PTSD and the Simoa biomarkers would be greater among *APOE* ε4 carriers compared to non-carriers.

The *National Institute on Aging* and *Alzheimer’s Association* have advanced a classification scheme for brain aging and Alzheimer’s disease biomarker research based on three continuous dimensions, termed *beta-amyloid deposition* (A), *pathologic tau* (T), and *neurodegeneration* (N) [7]. Known as the “ATN framework,” this system lends itself to latent variable modeling, which estimates the shared variance between indicators of a common dimensional construct, or factor (e.g., A, T, or N), and separates it from error variance in the indicators. This can be expected to increase power for association analyses relative to examining individual markers where true score variance and error variance are conflated. Theoretically, in the context of modelling associations among multiple correlated biomarkers, the resulting factors represent the broader biological construct underlying their covariation. Practically, this approach offers a method of data reduction that reproduces the observed relationships among multiple indicators using a smaller number of latent variables representing their commonalities. For the epigenome-wide association analyses reported here, this approach allowed us to distill the 5 Simoa biomarkers down to a smaller of number underlying factors, thereby reducing the study-wide multiple-testing burden.

This study was based on data from 849 ancestrally diverse individuals with a high prevalence of trauma exposure and PTSD spanning a wide age range with genome-wide DNA and DNA methylation (DNAm) data available for analysis. We began by performing factor analyses of the plasma Aβ40, Aβ42, GFAP, NfL and pTau-181 Simoa biomarkers the results of which yielded a two-factor solution with the Aβs loading on one factor and the three other markers loading on a second factor. We then performed epigenome-wide association analyses of each participant’s scores on the two factors with the aim of identifying novel epigenetic loci associated with levels of the ADRD biomarkers. Finally, using structural equation modeling, we evaluated (a) the influence of PTSD, age, *APOE* ε4 genotype and other relevant covariates on levels of the ATN factors, and (b) tested the mediating influence of the EWAS-significant DNAm loci on these associations.

## Methods

### Participants and procedures

Analyses were based on existing clinical, genetic, and biomarker data collected under research protocols led by investigators at the Behavioral Sciences Division of the VA National Center for PTSD [18]. Data for this report were from 849 individuals, including 580 US military veterans and a subset of 269 of their intimate partners, collectively ranging from 19 to 75 years of age. Clinical and demographic characteristics of the sample are listed in Table 1. Each of the original studies, and the research presented in this report, was reviewed and approved by the appropriate institutional review boards. In each study, participants had blood drawn for future genetic and biomarker assays. Blood was collected in EDTA tubes, centrifuged to separate plasma, serum, and buffy coat, then aliquoted and stored at − 80 °C until thawed for analysis. Participants also underwent psychiatric assessments using the Clinician-Administered PTSD Scale for *DSM-IV* or *DSM-5* (CAPS [19, 20]) and Structured Clinical Interview for *DSM-IV or DSM-5* (SCID [21, 22]), depending on the version of *DSM* in use at the time of study enrollment. For the CAPS, in addition to determining current diagnosis, frequency and intensity ratings of each symptom were summed to create a dimensional score reflecting current PTSD severity. We harmonized CAPS symptom severity across the two *DSM* versions by calculating each participant’s symptom severity as a percentage of the maximum possible severity score for the relevant version of the measure (yielding scores ranging from 0–1). All diagnostic interviews were video recorded. Inter-rater reliability was assessed for approximately 25% of participants; kappa for each diagnosis reported here was greater than 0.78. History of psychological trauma was assessed using the Traumatic Life Events Questionnaire (TLEQ) [23], an inventory of 23 different types of traumatic experiences that were coded as positive if the participant endorsed (a) exposure to the event, and (b) experiencing “intense fear, helplessness, or horror” when it happened. Additional details regarding the samples, clinical assessments, and inter-rater reliability are available in previous reports [24, 25]. Neurocognitive function was not formally assessed; however, participants were terminated from the procedure if, in the clinician’s judgment, cognitive impairment interfered with the participant’s ability to complete the procedures. Finally, current psychiatric medication use was assessed using a self-report checklist and then classified into four categories: (a) SSRI/SNRIs, (b) other antidepressants, (c) typical/atypical antipsychotic, (d) sedatives, hypnotics, anxiolytics.

**Table 1:** Sample descriptive statistics and bivariate correlations with the Simoa factor scores

	N or M	% or SD	Factor A	Factor TN
Age *(mean/SD)*	51.56	11.4	0.377***	0.528***
*APOE*ε4 carrier count			− 0.053	− 0.030
(One allele)	224	25.8		
(Two alleles)	20	2.2		
Sex			− 0.001	− 0.021
Male	523	61.6		
Female	326	38.4		
Self-identified Race				
White	574	67.6		
Black	95	11.2		
Asian/Pacific Islander	10	1.2		
Native American/Alaskan Native	64	7.5		
Self-identified Ethnicity				
Hispanic/Latino	123	14.5		
White, non-Hispanic	531	62.5		
Ancestral Principal Components				
PC1			0.107**	0.055
PC2			− 0.073*	− 0.079*
PC3			− 0.014	− 0.040
Veteran	580	68.3	0.005	− 0.017
Current Medications^a^				
SSRI/SNRIs	251	29.6	< 0.000	0.010
Other antidepressants	160	18.8	0.041	0.041
Typical/atypical antipsychotic	97	11.4	− 0.101**	− 0.106**
Sedatives, hypnotics, anxiolytics	138	17.4	− 0.018	− 0.020
Current PTSD Diagnosis^b^	311	36.6	− 0.025	− 0.098**
PTSD Symptom Severity *(mean/SD)*	0.31	0.2	− 0.062	− 0.133***
TLEQ *(mean/SD)*	1.73	1.94	0.115**	0.069
Current MDD Diagnosis	162	19.1	0.041	− 0.045
Current AUD Diagnosis	64	7.5	− 0.015	− 0.050
DNAm Smoking Score *(mean/SD)*	− 2.65	34.4	0.065	0.085*

The first two columns list the sample *n*s and percentages (unless specified as means/SDs). The second two columns list the bivariate correlations between each independent variable and factor scores derived from the CFA. These scores reflect each participant’s level on the latent Simoa variable. N = 849 except where otherwise noted

*PTSD* symptom severity was a standard score ranging from 0–1; *AUD* Alcohol Use Disorder; *DNAm* DNA methylation; *MDD* Major Depressive Disorder; *SSRI/SNRI* Selective serotonin reuptake inhibitor/serotonin and norepinephrine reuptake inhibitor; *TLEQ* Traumatic Life Events Questionnaire

^a^n = 789

^b^PTSD symptom severity was used in the analysis and clinician diagnosis is reported here for descriptive purposes

*p < 0.05; **p < 0.01; ***p < 0.001

### Genotype and DNA methylation data

Genetic data were generated using methods described in prior publications [18]. Briefly, DNA was isolated on a Qiagen AutoPure instrument with Qiagen reagents and samples normalized using PicoGreen assays (Invitrogen, Grand Island, NY, USA). Each DNA sample was run on an Illumina OMNI 2.5 microarray and scanned using an Illumina HiScan System (San Diego, CA, USA) according to the manufacturer’s protocol. Imputation was based on the Thousand Genomes Phase 3 reference panel [26]. Ancestry was determined using a pipeline [27, 28] that identified ancestral principal components using 100,000 randomly selected common single nucleotide polymorphisms (SNPs). *APOE* ε4 carrier status was called using the isoform-defining SNPs (*rs7412* and *rs429358*) which were well-imputed (*r*^2^ = 0.96 and 0.99, respectively). We used “best guess” imputed genotypes with a 90% confidence threshold for these SNPs to derive the *APOE* ε4 genotypes.

DNA methylation (DNAm) studies involve measurement of a methyl group on the DNA strand at a cytosine-phosphate-guanine (CpG) site. DNAm data were generated using methods described in prior publications [29]. In brief, DNAm was measured using the Illumina Infinium Methylation EPIC BeadChips. Zymo EZ-96 DNA Methylation Kits (D5004) were used to bisulfite-convert batched samples. DNA conversion was accomplished via PCR using DAPK1 primers (Zymo) followed by gel electrophoresis of PCR products. Bisulfite-modified DNA was then whole-genome amplified, hybridized to the BeadChips, single-base extended, and stained using the Automated Protocol for the Illumina Infinium HD Methylation Assay. Assignment of individuals to chip and chip positions were balanced based on PTSD diagnosis and sex. We applied a quality control (QC) pipeline developed by the Psychiatric Genomic Consortium-PTSD Workgroup [30] prior to analysis (and recently updated as described at https://github.com/PGC-PTSD-EWAS/EPIC_QC). Proportional white blood cell (WBC) estimates (CD8-T and CD4-T cells, natural killer cells, b-cells, monocytes) were calculated from the methylation data for use as covariates.

### Simoa markers

Simoa assays were performed at the Quanterix Accelerator Lab (Quanterix Corporation, Billerica, MA) using plasma samples. Samples were thawed and diluted per manufacturer’s specifications, centrifuged to remove particulates and debris, then pipetted into 96 well plates, diluted 4x, and run in duplicate. All markers were tested using the HD-1 Analyzer. Aβ40, Aβ42, GFAP, and NfL were assayed using the N4PE advantage kit (Quanterix Item #103,670). pTau181 was assessed using the pTau181 advantage v2 kit (Quanterix Item #103,714). We initially intended to include plasma total Tau, but preliminary analyses showed weak bivariate associations between this analyte and the other Simoa markers (*r*s < 0.2). Furthermore, unlike the other five markers which are primarily brain-derived, the Tau protein is also expressed in peripheral tissues [31], and a recent study estimated that only 20% of plasma total Tau originates in the brain [32]. Calibration was conducted with reference samples and QC procedures included evaluation of average enzyme per bead and coefficient of variation (CV). Samples that did not pass QC procedures were re-run, when possible, with the goal of minimizing freeze/thaw cycles. Samples were excluded (0–4.5%, varying by marker) if (a) the CV was > 25%, or (b) a duplicate was not available. Remaining concentrations were then multiplied by the dilution factor (× 4) prior to analysis. Results below the functional lower limit of quantification (fLLOQ) were set to the fLLOQ, and results above the functional upper limit of quantification (fULOQ) were set to the fULOQ. In total, data from 713 participants passed the DNAm and Simoa QC procedures, and of those, 704 also had genotype data.

### Data analyses

#### Exploratory and confirmatory factor analyses

Exploratory (EFA) and confirmatory factor analyses (CFA) of raw values for the five Simoa markers (Aβ40, Aβ42, GFAP, NfL, pTau181) were performed using Mplus v8.5 [33]. EFA is a method for identifying the structure and dimensionality underlying the covariation of set of variables when that structure is not known a priori. In EFA, models with different numbers of latent variables (factors) are compared to determine which model best accounts for the covarion among the variables. It is similar to principal components analysis except that EFA can distinguish between true score variability and error and separates these two sources of variance. CFA, in contrast, enables examination of the degree to which data fit a predefined, or a priori hypothesized, structure for the number of factors underlying the covariation of variables and loading of individual variables on each factor. In both approaches, the fit of the model to the data is evaluated using several commonly used fit indices including the root mean square error of approximation (RMSEA), standardized root mean square residual (SRMR), and the comparative fit and Tucker-Lewis fit indices (CFI and TLI, respectively). The Bayesian information criterion (BIC) can be used for evaluating the fit of competing models.

We began by conducting an EFA of the five markers in a random half of the sample and evaluated 1 and 2 factor solutions using geomin rotation. (With 5 markers, we could only evaluate 1- and 2-factor solutions due to the fact that a model with more factors would have been statistically under-identified.) The robust maximum likelihood estimator (MLR) was used in all analyses. The results of the best fitting (two-factor) EFA were then used to inform the structure of a CFA that we tested in the other random half of the sample. After identifying a good fitting two-factor CFA, we executed the same model in the full sample (*N* = 849), again evaluated fit, then saved those factor scores for use in the EWAS. The factor scores reflect each individual’s score on the latent variables (e.g., a higher score on the latent variable would account for higher values on the individual Simoa markers that load on that factor).

#### Epigenome-wide association analyses (EWAS)

We performed EWASs of scores from the two factors using linear models in the Bioconductor limma (Linear Models for Microarray Data) package [34], with the base 2 logit-transformed methylated proportion (known as an M values) as the response and the factor score as the predictor. Each EWAS included the following covariates: the top three ancestry principal components, age, sex, estimates of WBC proportions, a categorically coded batch variable representing the methylation project that each sample was assayed under, and a DNAm-based smoking score. The latter was based on effect-size estimates for the top-39 probes from a smoking EWAS [35] that we have previously shown to be an important covariate to include in DNAm association analyses [29]. We computed false discovery rate (FDR) corrected p values [36], also known as Q values, to control for multiple testing (denoted “padj”). Finally, we examined the genes corresponding to the top 500 sites from each EWAS for enrichment of specific gene ontology (GO) term categories using the gometh function from the R missMethyl package [37]. This function is an extension of the GOseq method [38] which explicitly models the relationship between the number of CpG sites assessed within a gene and the probability of that gene appearing within the target list.

#### Structural equation model

Structural equation modeling (SEM) is a multivariate analytic method for simultaneously estimating the strength of associations between latent variables (e.g., in this case, the Simoa factors) and other observed variables in a causal structure containing direct (regressive paths) and/or indirect (mediated) paths in a single analysis. It is like path analysis, but involves paths between latent variables, as opposed to between observed variables. As with the factor analyses, this was performed in Mplus. This model examined (a) the strength of associations between the independent variables (i.e., the demographic, psychiatric and *APOE* ε4 genotypes) and the dependent variables (i.e., the Simoa factors), (b) the influence of the independent variables on the M values from the CpG sites identified by the EWAS, and (c) the effects of covariates relevant to each variable in the model. In addition, given the role of DNAm in mediating effects of environmental factors on many forms of gene and protein expression, we also modeled the EWAS-significant CpG sites as mediators of the associations between the independent variables and the Simoa factors. Additional file 1: Fig. S1 illustrates full structural equation model including the latent variables, regressive paths, factor loadings, factor correlations, and covariates. Given the large number of parameters to be estimated and evidence that Type 1 error can become inflated in SEM analyses [39], we utilized a conservative *p* value threshold of 0.001 for all direct regressive paths in the model. Finally, for significant CpG associations, we also evaluated the significance of the indirect (mediated) effect of the IV on the DV via the CpG loci using the “model indirect” command in Mplus which computed the products of (a) the effect of each IV on each CpG, and (b) the effect of each CpG on the Simoa factor, along with the *p* values for each indirect path.

## Results

### Exploratory and confirmatory factor analyses

Descriptive statistics for, and bivariate correlations between, the five Simoa markers (Aβ40, Aβ42, GFAP, NfL pTau181) are listed in Table 2. The EFA in a random half of the sample yielded excellent model fit for a 2-factor solution that was superior to the 1-factor model (see Table 3). In the two-factor EFA solution, Aβ42 (*λ* = 0.76) and Aβ40 (*λ* = 0.72) loaded significantly on one factor, while pTau181 (*λ* = 0.50), GFAP (*λ* = 0.56), and NfL (*λ* = 0.72) loaded significantly on the second factor. The cross-loadings were all small and nonsignificant (λs = − 0.009 to.19, *p*s > 0.05).

**Table 2:** Descriptive statistics and correlations between the Simoa markers

	Aβ40	Aβ42	GFAP	NfL
Aβ42	0.632	–	–	–
GFAP	0.374	0.295	–	–
NFL	0.384	0.270	0.414	–
pTau^181^	0.204	0.135	0.227	0.294
pTau^181^	Aβ40	Aβ42	GFAP	NfL
1.70 (0.84)	91.69 (18.52)	6.78 (1.53)	70.92 (34.38)	12.28 (7.44)

pTau^181^	Aβ40	Aβ42	GFAP	NfL
1.70 (0.84)	91.69 (18.52)	6.78 (1.53)	70.92 (34.38)	12.28 (7.44)

*n* = 816 due to some pairwise comparisons having missing data. All *p* values < 0.001

Bottom row lists the means and (SDs) for each marker in pg/ml units

**Table 3:** Model fit of the exploratory and confirmatory factor analyses

	Model	χ^2^	*df*	*p*	RMSEA	SRMR	CFI	TLI	BIC
EFAs	1 Factor (1st half sample)	51.87	5	< 0.001	0.15	0.07	0.85	0.70	13,120
	2 Factor (1st half sample)	0.004	1	0.948	< 0.001	< 0.001	1.00	1.00	13,085
CFAs	2 Factor (2nd half sample)	4.36	4	0.359	0.015	0.018	0.999	0.997	12,884
	2 Factor (full sample)	5.41	4	0.248	0.020	0.014	0.998	0.994	25,897

*n* = 849

*EFA* exploratory factor analysis; *CFA* confirmatory factor analysis; *RMSEA* root mean square error of approximation; *SRMR* standardized root mean square residual; *BIC* Bayesian information criterion; *CFI* comparative fit index; *TLI* Tucker-Lewis Index

Based on this, we then tested a CFA in the second random half of the sample (*n* = 421) in which Aβ40 and Aβ42 were set to load on one factor (that we term “*Factor A*,” i.e., “A” from the “ATN” framework) and pTau181, GFAP, and NFL on a second factor (that we term “*Factor TN*”). This model fit the data well (Table 3) with all indicators loading on their respective factors at the *p* < 0.001 level. This model also yielded the lowest (i.e., best) BIC value compared to the EFA models. We then ran the 2-factor CFA in the full sample (*n* = 849) and found that this model also fit well in the full cohort with each indicator again loading significantly on its respective factor at the *p* < 0.001 level. The two factors were correlated at *r* = 0.62 (*p* < 0.001). Finally, we saved the factor scores from this analysis for use in the EWASs. Scatterplots of the associations between each factor, their indicators, and between the indicators themselves, are shown in Additional file 1: Figs. S2, S3.

### Epigenome-wide association analyses

**Fig. 1:**
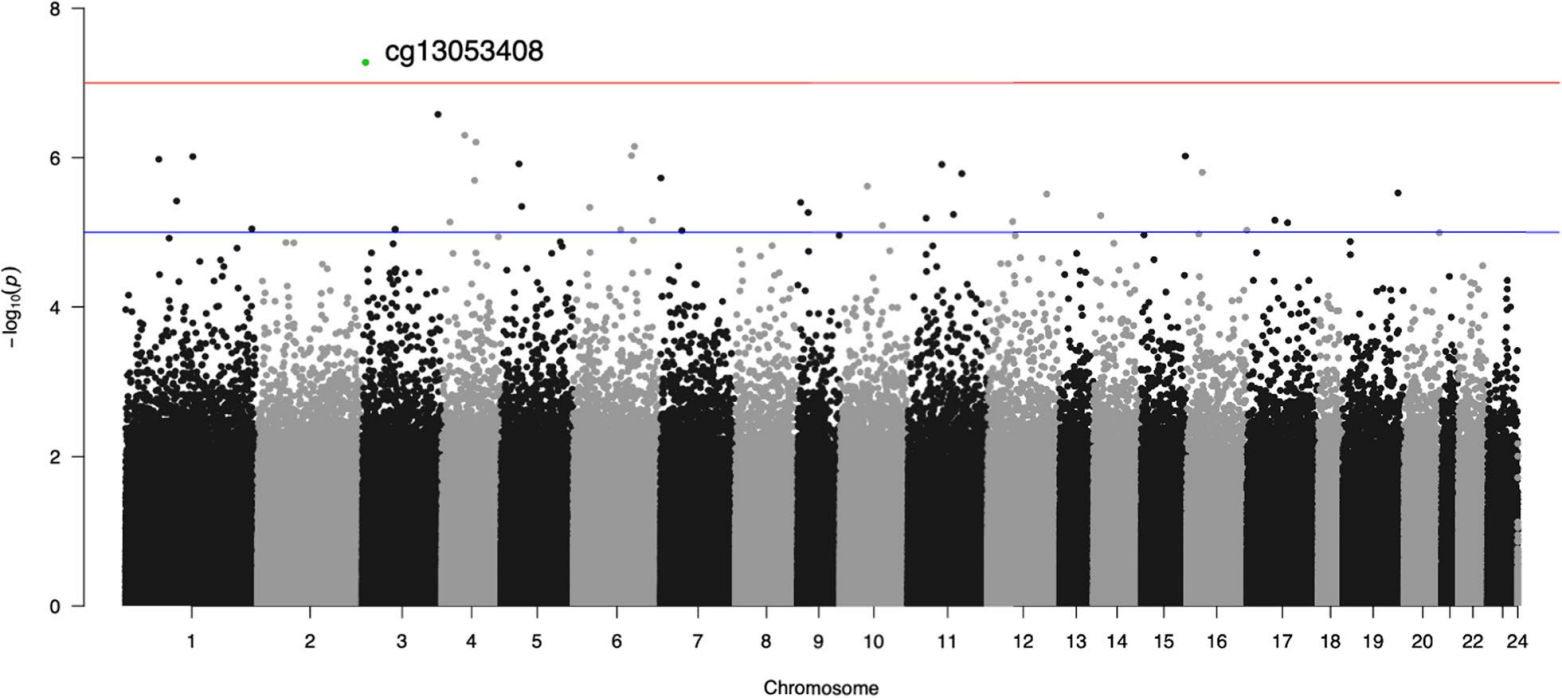
Manhattan plot of the Factor A EWAS. The x-axis depicts chromosomes and the location of each CpG site across the genome. The y-axis is the -log10 of the p value for the association with levels of Factor A Simoa markers defined by Aβ40 and Aβ42. Each dot represents a CpG site. The red line indicates the level for epigenome‐wide statistical significance (*p* = 1 × 10^–7^) and the blue line the level for suggestive significance (*p* = 1 × 10^–5^). The EWAS-significant locus is highlighted in green

**Fig. 2:**
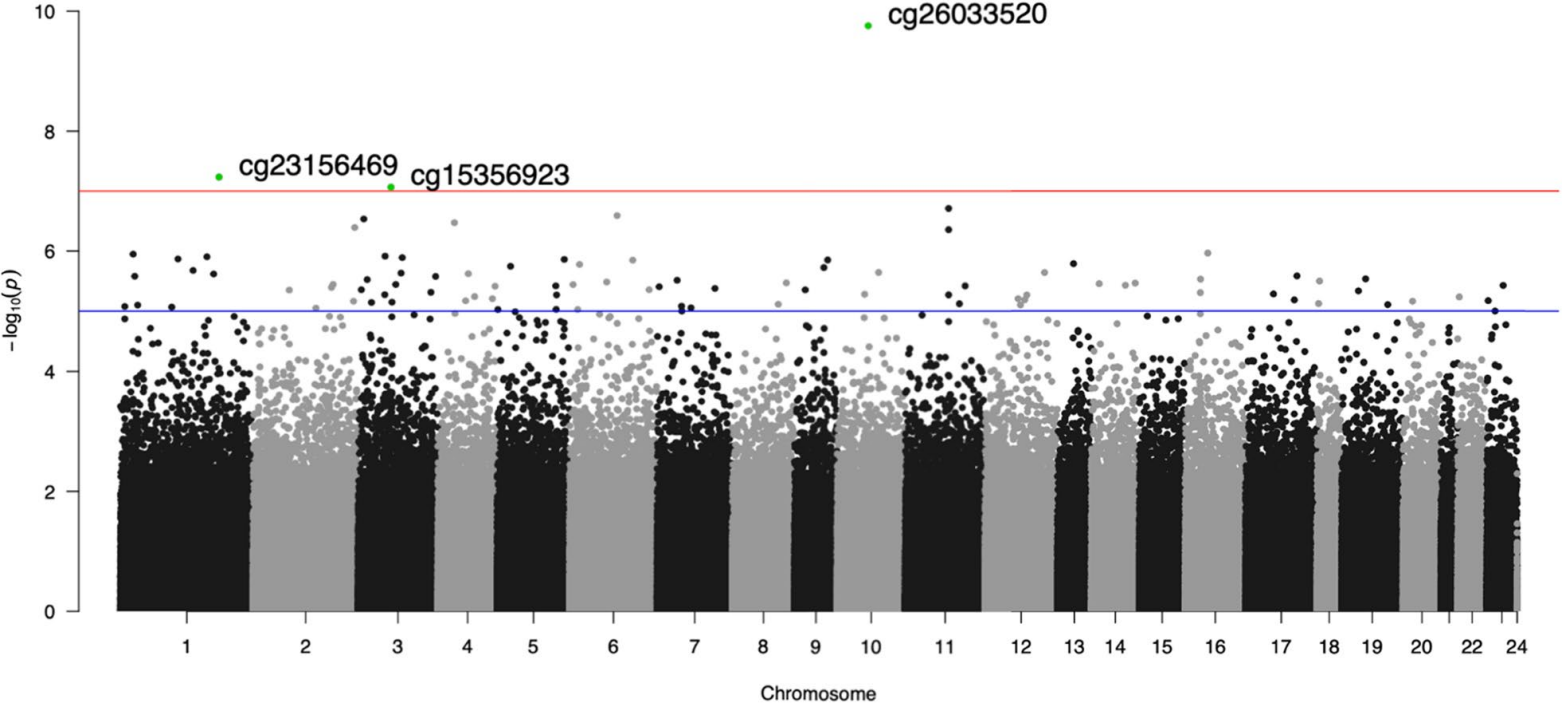
Manhattan plot of the Factor TN EWAS with the three EWAS-significant loci highlighted in green

Lambdas for the EWASs indicated modest inflation (Factor TN = 1.201; Factor A = 1.249). After applying an FDR correction across the two analyses (total # of comparisons = 1,605,282), 3 loci were identified as significantly associated with Factor TN (cg26033520, cg23156469, cg15356923) and one was significantly associated with Factor A (cg13053408). See Table 4 for the EWAS-significant loci, Figs. 1 and 2 for the EWAS Manhattan plots, and Additional file 1: Figs. S4, S5 for the EWAS QQ plots. GO term enrichment analysis of the top 500 most significant probes from each EWAS yielded no FDR significant GO terms. The top-50 probes from each EWAS are listed in Additional file 1: Tables S1, S2. A comparison of the list of *p* < 0.001 significant probes from the two EWASs showed that 12.9 percent of these loci overlapped. Full summary statistics from each EWAS are available in the Additional files 2 and 3.

**Table 4:** Epigenome-wide significant CpG sites by factor

Factor	CpG	logFC	*p* value	FDR	Gene
T/N	cg26033520	− 0.4147	1.75e^−10^	0.0003	*ASCC1* (nearby)
	cg23156469	− 0.1939	5.84e^−08^	0.0312	*FAM20B*
	cg15356923	0.2150	8.59e^−08^	0.0345	*FAM19A4 (aka TAFA4)*
A	cg13053408	− 0.0027	5.30e^−08^	0.0312	*C3orf24 (aka FANCD20S)*

*n* = 704 (lower due to samples failing the GWAS, EWAS or SIMOA QC pipeline). Covariates were age, sex, ancestry PCs 1–3, estimated cell proportions (CD4T, CD8T, B cell, NK and Mono), DNAm-based smoking score, and laboratory analysis batch. FDR correction was based on the total number of probes examined across the two EWASs (1,605,282 probes total)

### Structural equation model

**Fig. 3:**
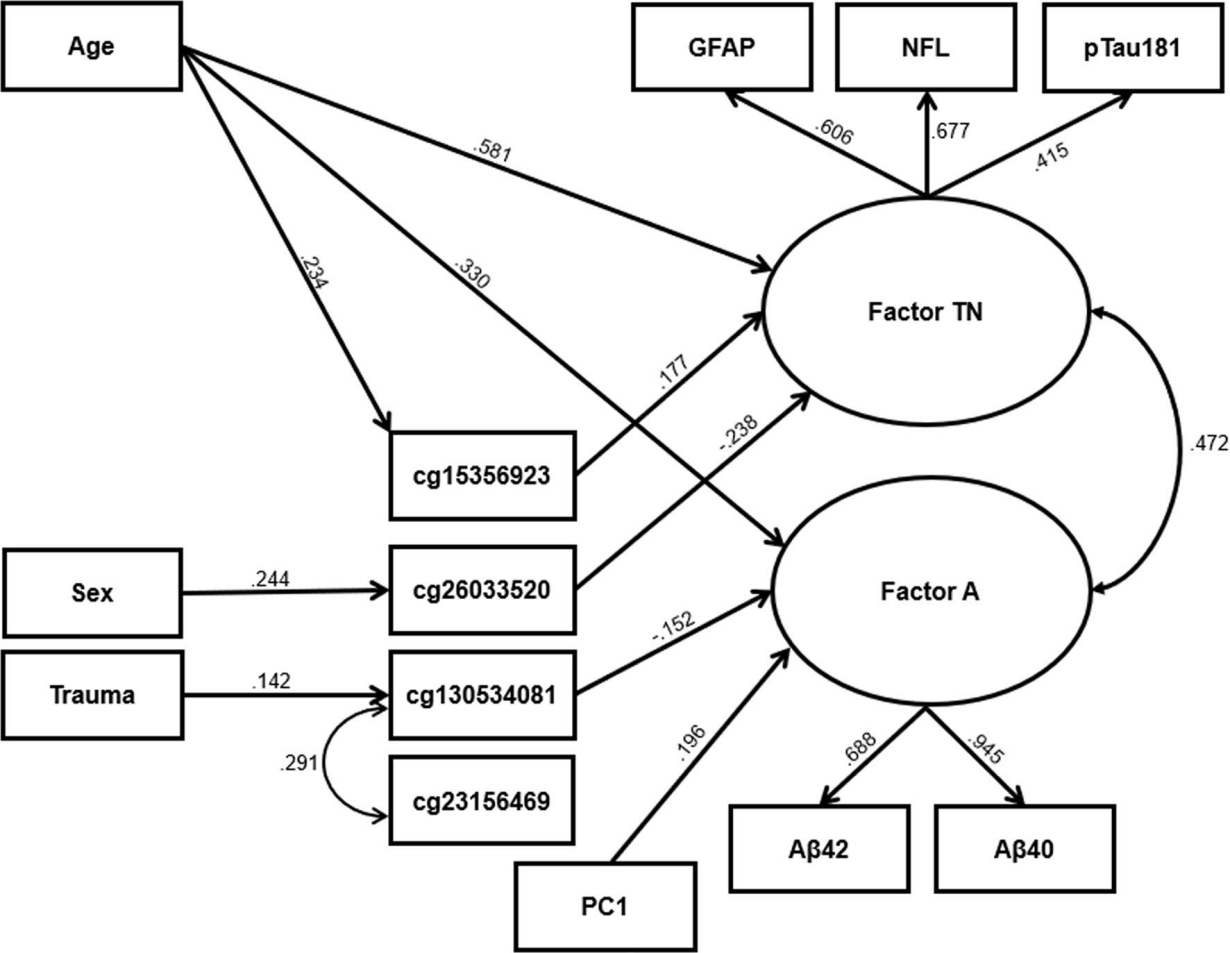
Diagram depicting the significant direct paths (*p*s < 0.001) and associated variables from the SEM results. The Simoa latent variables are represented as circles with the loadings of each indicator shown as well as the correlation between the two factors. Age was the only variable in the model that showed significant (*p* < 0.001) direct effects on both variables. PC1 was positively associated with Factor A. The EWAS-significant CpG sites were evaluated as mediators of the association between the psychiatric and demographic variables and the latent variables. Significant indirect effects were found for **a** the effect of age on Factor TN via *cg15356923*, **b** the effect of sex on Factor TN via *cg26033520*, and **c** the effect of lifetime trauma count on Factor A via *cg130534081*

The bivariate correlations between the two Simoa factors and each of the clinical, genetic, and demographic variables are listed in Table 1. Table 5 lists the bivariate correlations between the primary independent variables and each individual Simoa marker. Figure 3 shows the significant paths and parameter estimates (*p*s < 0.001) from the SEM. (Full results are available in Additional file 1: Table S3; scatterplots of the associations between the PCs are depicted in Additional file 1: Fig. S6). Fit statistics showed adequate model fit (*χ*^2^ = 160.09, *df* = 68, *p* < 0.001; RMSEA = 0.045, CFI = 0.93, TLI = 0.83, SRMR = 0.03). In total, the model explained 54% of the variance in the Factor TN and 23% of the variance in Factor A. As shown in Fig. 3, age showed the strongest associations with both factors and was more strongly associated with Factor TN than Factor A. To test this difference empirically, we ran a follow-up model in which these age paths were held to equivalent and found that doing so significantly degraded model fit (Δ *χ*^2^ = 87.32, Δ df = 1,* p* = 9.23e^−21^). Age was also significantly associated with *cg15356923* and the indirect effect of age on Factor TN via this DNAm locus was significant (*β* = 0.041, *p* = 0.005). Sex was positively associated with *cg26033520* (greater for females) and the indirect effect of sex on Factor TN via this locus was significant (*β* = − 0.058, *p* < 0.001). Lifetime trauma count was positively associated with *cg130534081* and the indirect effect of trauma on Factor A via this locus was also significant (*β* = 0.022, *p* = 0.008). We also observed a significant positive residual correlation between DNAm levels at *cg130534081* and *cg23156469*, implying additional covariation among these loci beyond that which could be attributed to the shared predictors of these loci. *APOE* ε4, PTSD, and their interaction were not significantly associated with any of the CpGs or either factor (*p*s > 0.001).

**Table 5:** Bivariate correlations between the primary independent variables and each Simoa marker

	Age	*APOE* ε4	PTSD	Sex	Trauma
Aβ40	0.362***	− 0.034	− 0.034	− 0.002	0.120**
Aβ42	0.172***	− 0.103**	− 0.066	0.026	0.063
GFAP	0.412***	− 0.011	− 0.128***	0.051	0.137***
NfL	0.438***	− 0.028	− 0.105**	− 0.040	− 0.040
pTau^181^	0.239***	0.042	− 0.013	− 0.141***	− 0.049

*p < 0.05; **p < 0.01; ***p < 0.001

The SEM analysis also revealed a significant positive association between Factor A and PC1 (*β* = 0.196, *p* < 0.001) controlling for age and the other variables in the SEM, indicating that individuals with a greater proportion of African ancestry had lower levels of Aβs relative to those of European ancestry. This difference was also significant in the categorical ancestry classification based on the genotype data (African vs. European ancestry clusters; *p* = 0.004) and for self-identified Black participants compared to self-identified White individuals (*p* = 0.012). All of these differences were significant when controlling for age. Finally, as a sensitivity analysis, we ran the full SEM with the addition of the four classes of psychiatric medications and found no significant associations between current medication use and either factor (all *p*s > 0.001).

## Discussion

Research on blood-based biomarkers of ADRD is rapidly advancing, yet relatively little is known about how these markers covary, or what influence various genetic, epigenetic, demographic, and psychiatric factors have on their levels. In this study, we used factor analyses to identify two dimensions that explained the covariation among Aβ40, Aβ42, GFAP, NfL and pTau-181. Drawing from the *National Institute on Aging* and *Alzheimer’s Association* “ATN” research framework, we termed the first factor, defined by Aβ40 and Aβ42, “Factor A”. GFAP, NfL and pTau-181 loaded on a second factor that we accordingly termed “Factor TN.” Next, we performed an EWAS of each factor, the results of which identified 4 DNAm loci that survived multiple-testing correction across the two analyses. The most strongly associated CpG from the Factor TN EWAS, *cg26033520*, was located in the vicinity of the *Activating Signal Cointegrator Complex Subunit 1* gene (*ASCC1). ASCC1* transcribes a protein that plays a role in gene transactivation via its effects on several transcription factors. This locus was one of two EWAS-significant CpGs identified in a recent study of blood DNA methylation in Parkinson’s disease patients, with the same direction of association [40]. In a subsequent meta-analysis of epigenome-wide associations across Parkinson’s disease, Alzheimer’s disease, and amyotrophic lateral sclerosis (ALS), this CpG also showed significant shared effects across all three disorders [41]. ASCC1 exerts an inhibitory effect on *nuclear factor kappa-B* (NF-kB) [42], a well-established inflammatory transcription factor implicated in tau pathology, glutamate excitotoxicity, and reactive microglia and astrocytes [43]. The top-hit from the Nabais et al. [41] EWAS, *cg03546163* in *FKBP5*, was also among the top-50 most highly associated probes in our Factor TN results.

The other two EWAS-significant loci from the Factor TN analysis were *cg23156469* and *cg15356923*, located in *FAM20B* and *FAM19A4* (aka *TAFA4*), respectively. *FAM20B* transcribes a kinase that phosphorylates xylose residues and triggers the synthesis of peptidoglycan, the main component of the cell wall in most bacteria. Though the literature on this is gene is very limited, a SNP in *FAM20B* (*rs4652345*) was the 16th most strongly associated locus in a GWAS of individuals with polygenic risk extremes for Alzheimer's disease in the UK Biobank [44]. *FAM19A4 (*aka *TAFA4)*, on the other hand, transcribes a well-known neurokine that has been implicated in the regulation of immune responses and pain signaling within the nervous system [45].

The Factor A analysis identified one EWAS-significant locus, *cg13053408*, located in *FANCD2OS* (i.e., “*FANCD2* opposite strand” aka C3orf24). As an opposite strand gene (i.e., located on the antisense or noncoding strand of DNA), this region of DNA serves as the template for making *FANCD2* messenger RNA. FANC2D has been identified as a mediator of the effect of the amyloid precursor protein fragment *APP intracellular C-terminal domain* on FOXO3 [46] which has a well-established role in aging and many age-related disease processes [47, 48] including Amyloid-β induced astrocytosis and astrocyte death [49]. *FANCD2OS* overexpression has also been shown to modulate expression of the steroidogenic acute regulatory protein (STAR1 or STARD1) [50]. STAR1 is an intracellular cholesterol carrier that has been observed to be elevated in the cortex of Alzheimer’s disease and Down syndrome patients and correlated with Aβ42 deposition in regions of the hippocampus [51]. Also relevant to our findings, a pair of SNPs in *FANCD2* and *FANC2DOS* (*rs1552244* & *rs9849434*) were among the top-25 hits from an early Alzheimer’s disease GWAS meta-analysis [52] (albeit not replicated in more recent studies with larger Ns).

We then submitted the EWAS-significant DNAm values to an SEM analysis that evaluated these CpG sites as possible mediators of associations between age, sex, trauma history, PTSD, *APOE* ε4 genotypes and the two Simoa factors. Age was positively associated with both factors and significantly more so with Factor TN than Factor A. These findings align with a rapidly growing body of research showing that these markers are present in plasma of adults across the lifespan, trending gradually higher through early and middle adulthood, and then increasing exponentially in old age [12–14]. Mediation analyses identified three small but statistically significant indirect effects of age and sex on Factor TN, and number of lifetime traumas on Factor A, that were mediated by the CpGs. Though interesting and worthy of future examination, given the small size of the effects and novelty of the findings we are hesitant to over-interpret those results.

Genotype-determined ancestry PCs were included as covariates of the two Simoa factors and each CpG locus in the EWAS and SEM. The SEM revealed a significant positive association between Factor A and PC1 indicating that individuals with a greater proportion of genotype-determined African ancestry tended to have lower levels of Aβ40 and Aβ42. Similarly, self-identified Black participants showed significantly lower levels of Factor A than self-identified White individuals. Though not an a priori focus of the study, these findings are consistent with results of recent studies that examined racial differences in levels of plasma Aβs and brain amyloid levels. For example, Hajjar et al. [53] examined associations between self-identified race and genetic ancestry in 300 Black and 429 White individuals, the majority of whom had mild cognitive impairment and were over 60 years of age. Results showed that Black participants had significantly lower unadjusted levels of Aβ40 compared to White individuals and, as in our study, the percentage of African ancestry in the sample was negatively correlated with Aβ40 levels. Similarly, Deters et al. [54] examined racial differences in positron emission tomography (PET) measured amyloid in cognitively normal older adults and found that Black individuals had lower amyloid levels compared to White participants and this difference was enhanced as a function of the participant’s proportion of African ancestry. Finally, in an earlier study of nearly one-thousand adults in their seventies approximately 50% of whom were Black, Metti et al. [55] found significantly lower levels of plasma Aβ40 and Aβ42 among self-identified Black compared to White individuals. In sum, results of this study add to a growing body of findings suggesting that there are important racial and ancestral differences in age- and AD-related biomarkers that may be relevant for advancing understanding of racial differences in the rates of ADRD reported in large-scale epidemiological and cohort studies [3, 56].

We had hoped that this study would shed new light on possible mechanisms by which PTSD confers risk for dementia but hypotheses for our primary variables of interest, *APOE* ε4, PTSD (and their interaction) were not supported. Specifically, none of the direct effects of *APOE* ε4, PTSD, or the *APOE* ε4 x PTSD interaction term on the Simoa factors met our SEM criterion for statistical significance (*p* < 0.001). Rather, and contrary to our hypothesis that PTSD would be associated with elevated levels of ATN biomarkers, the SEM revealed a negative correlation between PTSD and Factor TN that fell just short of our multiple testing threshold (*β* = − 0.140, *p* = 0.003) controlling for age, sex, number of lifetime traumas, *APOE* ε4, the *APOE* ε4 x PTSD interaction term, the ancestry PCs, a DNAm-based smoking score, and effects of the four EWAS-significant probes. The simple bivariate correlation between PTSD severity and the TN factor scores showed a similar association (*r* = − 0.133, p < 0.001) driven primarily by reduced levels of GFAP in individuals with more severe PTSD symptoms (*r* = − 0.128, *p* < 0.001). We also noted that antipsychotic medication use was associated with reduced levels of both Simoa factors, but our sensitivity analyses showed that the negative PTSD effect remained significant and unchanged with medication use in the model.

Though unexpected and opposite in direction of what we hypothesized, the finding of reduced GFAP in association with PTSD is not without precedent. Pierce et al. [57] examined a panel of ATN plasma biomarkers in a younger cohort of 550 post-9/11 veterans (from the same VA medical center, but independent of the participants evaluated in this study) and also reported a modest negative correlation between PTSD severity and GFAP controlling for age and sex (*r* = − 0.10, *p* < 0.05). Kulbe et al. [58] conducted a prospective observational study of 1,143 emergency department patients with mild TBI evaluated within 24 h of injury then re-assessed 6 months later and found that day-of-injury plasma GFAP levels were significantly lower among those with a PTSD diagnosis at follow-up. Finally, Natale et al. [59] examined a sample of 1,520 World Trade Center responders and also found that PTSD was associated with reduced levels of plasma GFAP. These authors offered several hypothetical explanations for this finding and while the association appears worthy of future investigation, its biological significance remains unclear.

More generally, previous studies on the association between PTSD and ATN biomarkers have shown mixed and largely null results. For example, in arguably the most methodologically rigorous prior study on this topic, Weiner et al. [60] examined 289 non-demented Vietnam-era veterans with traumatic brain injury (TBI) and/or PTSD who underwent cognitive testing, cerebrospinal fluid collection, and Aβ and tau PET scans. Results showed that compared to controls, veterans with histories of TBI and PTSD were more likely to have mild cognitive impairment and lower mental status scores but there were no differences between groups in any of the ATN biomarkers examined. The apparent discrepancy between findings from large cohort and epidemiological studies on PTSD and risk for ADRD, and studies of the association between PTSD and ATN biomarkers, may be attributable to a variety of issues including (a) the possibility that the PTSD-dementia association is mediated by medical comorbidities such as cardiovascular disease and diabetes, (b) that this association is driven by non-AD specific neuropathology such as vascular or other forms of dementia, and/or (c) inherent differences between the clinical diagnosis of dementia in medical records versus the biological processes indexed by specific ATN biomarkers.

The findings of this study should be evaluated in the context of several limitations. Most notably, the study was based on cross-sectional archival data from projects that did not include neurocognitive measures or neuroimaging data and most of our participants were well below the age at which dementia is normally manifested. It is conceivable that significant effects of PTSD on brain age- and AD-associated biomarkers would be observed in older cohorts and/or individuals with PTSD and mild cognitive impairment or dementia. On the other hand, the study featured a large and ancestrally diverse sample with genome-wide SNP and DNAm data, and 5 Simoa markers indexing essential components of the ATN biomarker framework. Findings identified (a) novel and biologically plausible epigenetic associations with the factors that underlie the covariation of these markers which should inform future efforts to identify epigenetic loci reliably associated with ADRD and its biomarkers, and (b) robust age and race/ancestral associations that will be essential to consider in future efforts to develop the clinical and diagnostic applications of these tests.

### Supplementary Information

**Additional file 1. Supplementary Figure 1.** Illustration of the Full Structural Equation Model

**Additional file 2.** Supplementary Data Files.

**Additional file 3.** Supplementary Data Files.

## Data Availability

Qualified investigators can gain access to study data by contacting Dr. Miller and developing a Data Use Agreement with the PTSD Genetics Data Repository (Miller, PI).
