# Supplementary material for: An EWAS of dementia biomarkers and their associations with age, African ancestry, and PTSD
Source: Clin Epigenetics. 2024 Mar 2;16:38. doi: 10.1186/s13148-024-01649-3 (PMC10908031; doi:10.1186/s13148-024-01649-3)
Supplement: Supplementary file 1 — Additional file 1. Supplementary Figure 1. Illustration of the Full Structural Equation Model [file 13148_2024_1649_MOESM1_ESM.docx]

**Supplementary Figure 1. Illustration of the Full Structural Equation Model**


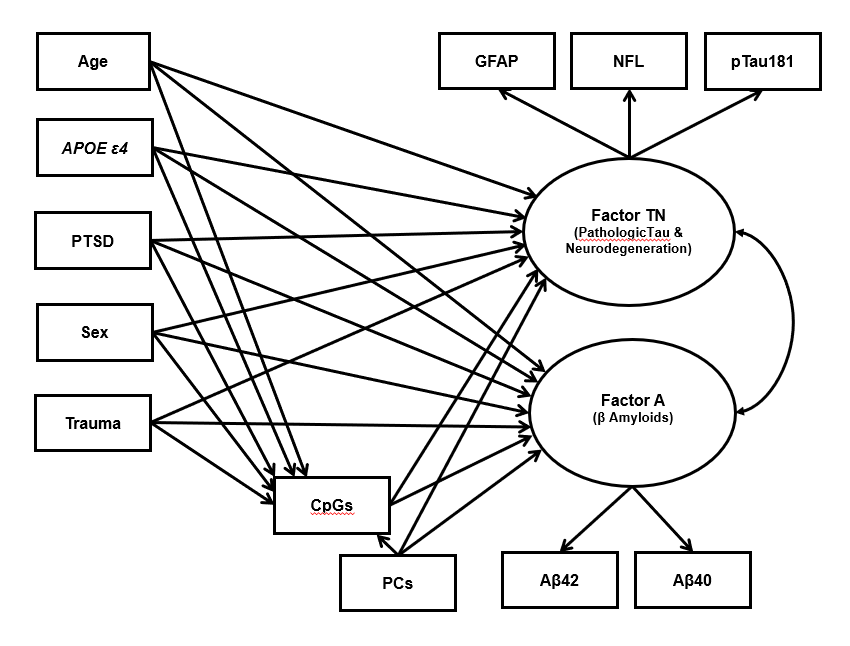


**Figure Note.** Illustration of the regressive paths, factor loadings, and factor correlations included in the SEM. To enhance legibility, the figure combines the 4 EWAS significant CpGs into one box and the 3 genetic ancestry PCs into another box. Not depicted are (a) the *APOE* ε4 x PTSD interaction term, and (b) the estimated WBC proportions and DNAm smoking score that were included as covariates of each CpG.

**Supplementary Figure 2. Scatterplots of associations between Factor A and its indicators**


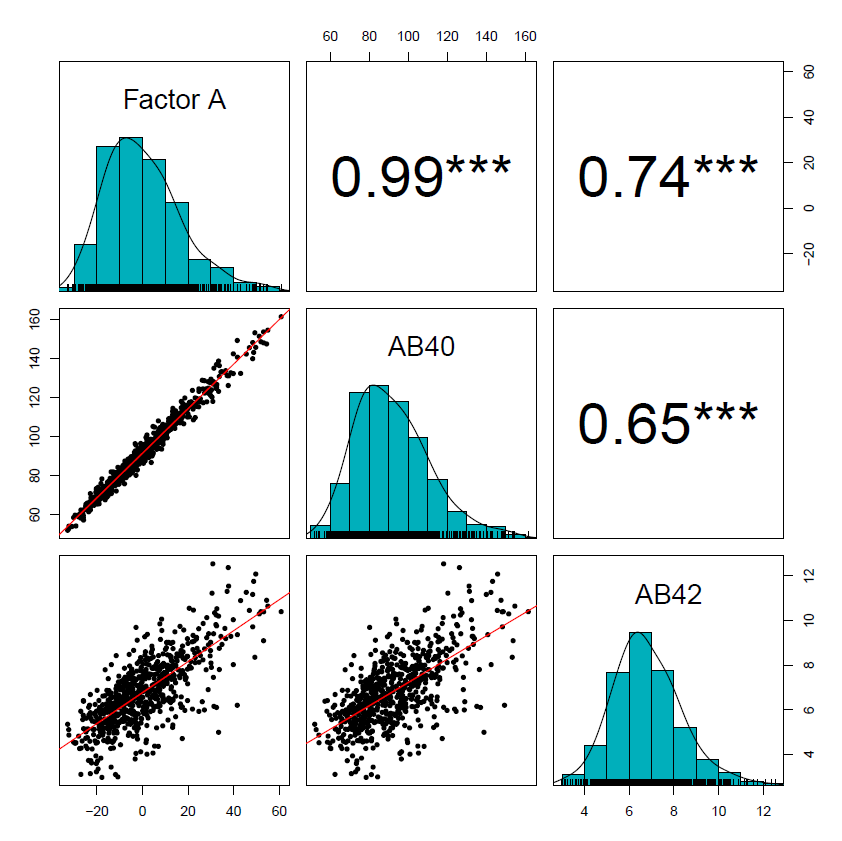


Note: Numbers above the diagonal are pairwise pearson correlation coefficients. *** = *p* < .001

**Supplementary Figure 3. Scatterplots of associations between Factor TN and its indicators**


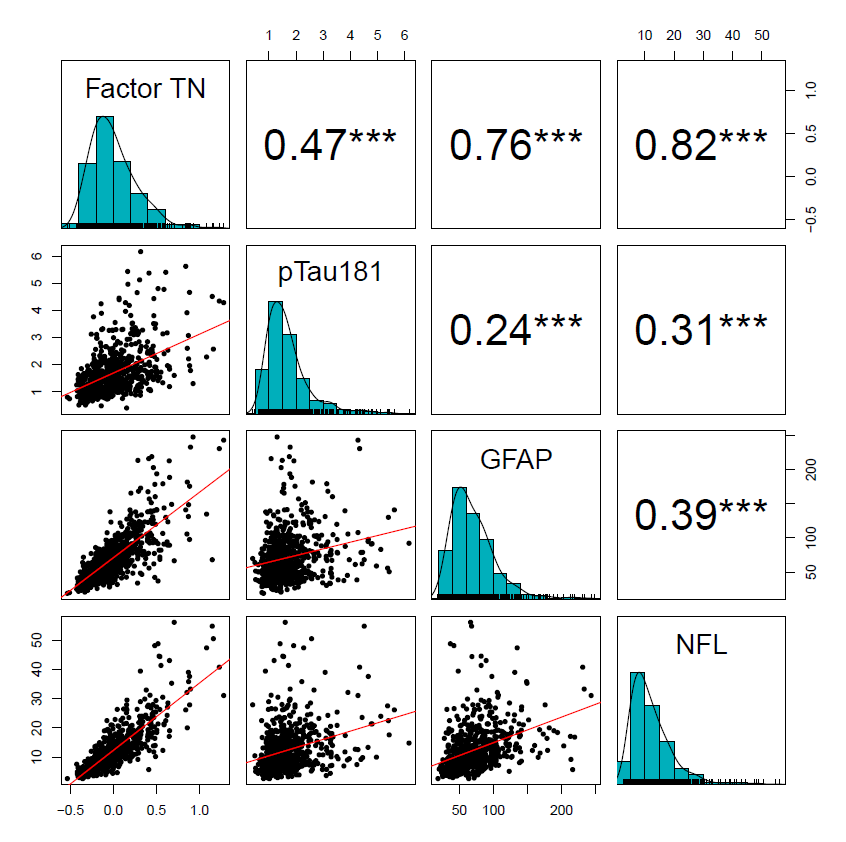


**Supplementary Figure 4. QQ plot for the Factor TN EWAS**


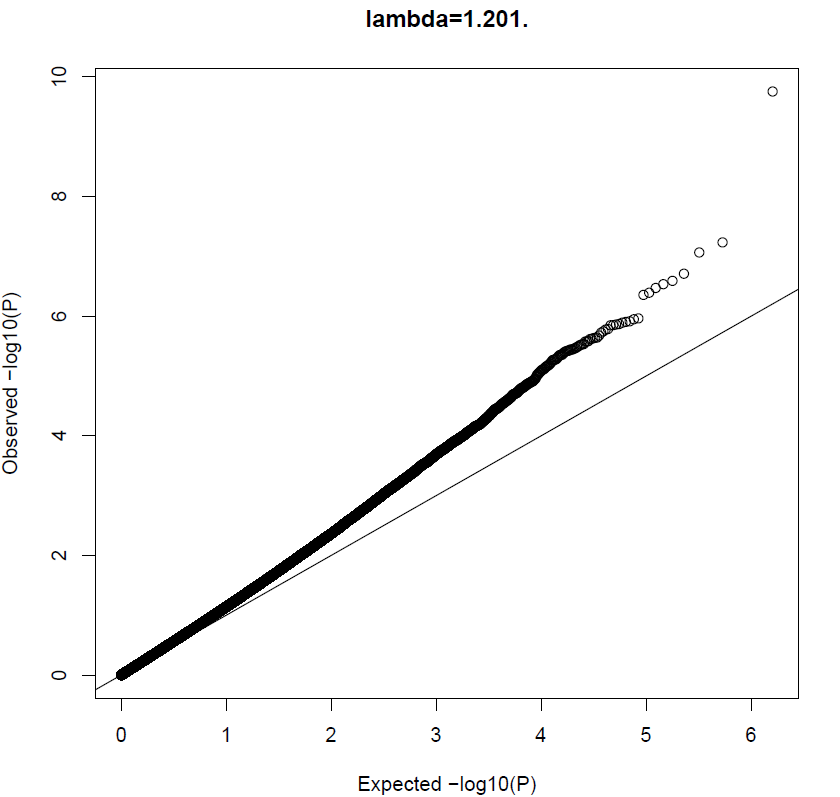


**Supplementary Figure 5. QQ plot for the Factor A EWAS**


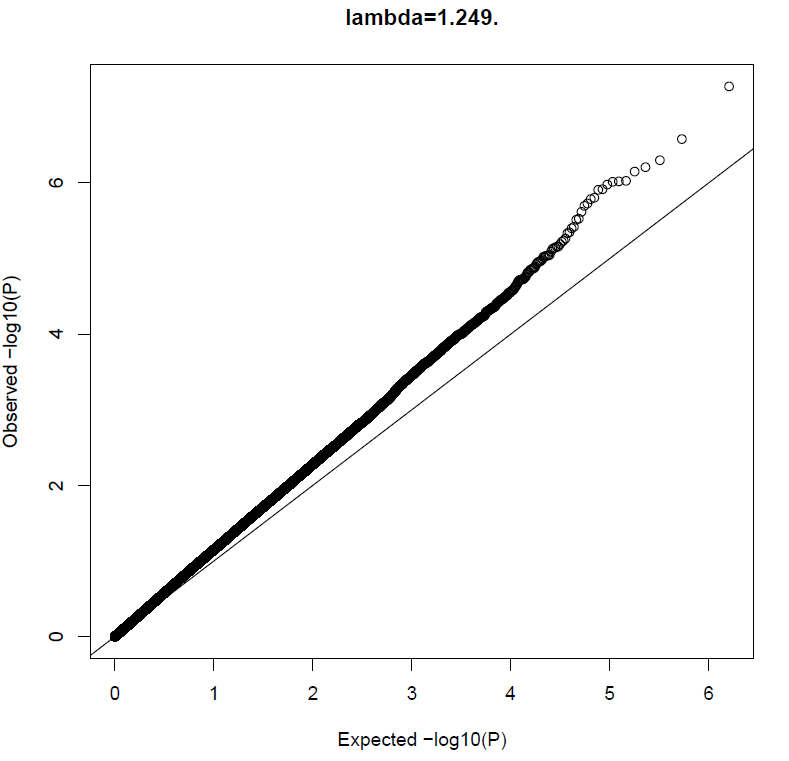


**Supplementary Figure 6. Pairwise Scatterplots of the first three Ancestry PCs**


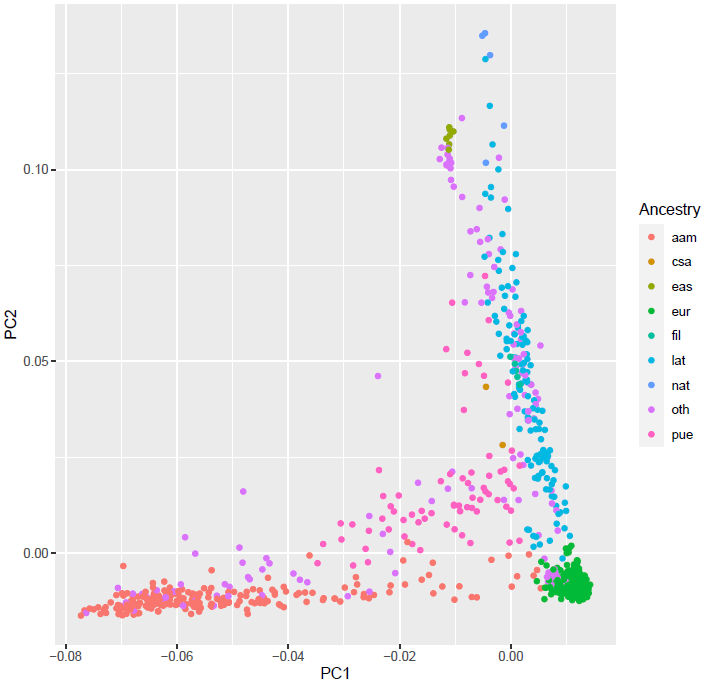

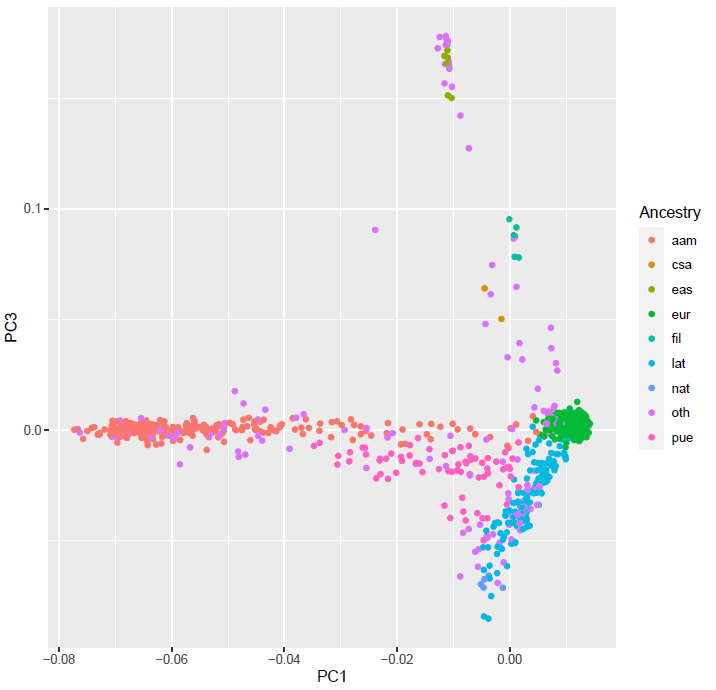


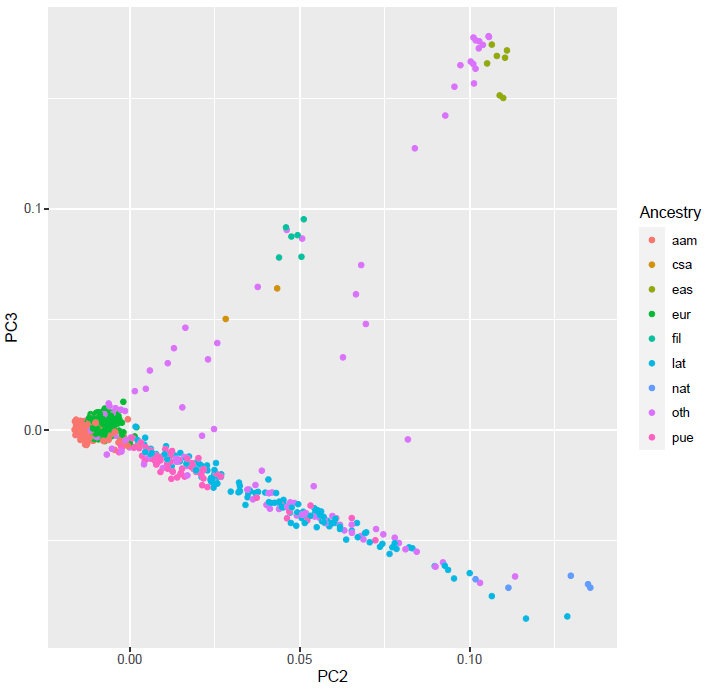


**Supplementary Table 1: Top 50 Associations from the Factor A EWAS**

| CpG | Beta | *p*-value | FDR | Chr | Position | Gene |
| --- | --- | --- | --- | --- | --- | --- |
| cg13053408 | -0.002706458 | 5.29991E-08 | 0.031229379 | 3 | 10104645 | *C3orf24; FANCD2OS* |
| cg17334836 | -0.002789916 | 2.63456E-07 | 0.058489366 | 3 | 194055399 | Intergenic |
| cg26504237 | 0.003665108 | 5.01437E-07 | 0.067078932 | 4 | 54230158 | *PDGFRA* |
| cg10923761 | -0.002561984 | 6.20019E-07 | 0.076561985 | 4 | 98496108 | *TSPAN5* |
| cg20751430 | -0.005531112 | 7.08462E-07 | 0.078547425 | 6 | 111760053 | *FYN* |
| cg15336269 | 0.003182164 | 9.40069E-07 | 0.078547425 | 6 | 106361361 | Intergenic |
| cg12954230 | 0.002289939 | 9.54293E-07 | 0.078547425 | 15 | 100342025 | *ADAMTS17* |
| cg27610536 | -0.004187047 | 9.68534E-07 | 0.078547425 | 1 | 95655528 | Intergenic |
| cg12888632 | 0.002489699 | 1.05239E-06 | 0.078547425 | 1 | 29283552 | *PTPRU* |
| cg08874888 | -0.002226016 | 1.21277E-06 | 0.078547425 | 5 | 53090657 | *ITGA2* |
| cg15598662 | 0.003366751 | 1.23579E-06 | 0.078547425 | 11 | 61815417 | *MIR1908;FADS1* |
| cg16712866 | -0.002270366 | 1.5759E-06 | 0.081317806 | 16 | 12265439 | *SNX29* |
| cg10790186 | -0.002932074 | 1.63727E-06 | 0.081317806 | 11 | 77993940 | *INTS4* |
| cg27640234 | -0.002865347 | 1.87392E-06 | 0.084031172 | 7 | 223444 | *FAM20C* |
| cg21390350 | 0.003155065 | 2.01605E-06 | 0.087468532 | 4 | 89111653 | *TIGD2* |
| cg17257757 | -0.002563973 | 2.41643E-06 | 0.088160165 | 10 | 68297547 | *PBLD* |
| cg15234312 | 0.002666047 | 2.97406E-06 | 0.093248119 | 19 | 53869069 | *MYADM* |
| cg12802812 | -0.002362069 | 3.07868E-06 | 0.093248119 | 12 | 120554339 | *RNF10* |
| cg20652681 | -0.002484153 | 3.81838E-06 | 0.093944309 | 1 | 52631599 | Intergenic |
| cg03503980 | -0.001975483 | 3.98497E-06 | 0.094073498 | 9 | 14850344 | *FREM1* |
| cg03942934 | -0.001779434 | 4.51503E-06 | 0.096638636 | 5 | 59357121 | *PDE4D* |
| cg23490638 | 0.003594489 | 4.63842E-06 | 0.096869861 | 6 | 28589689 | Intergenic |
| cg12325507 | 0.003951045 | 5.43883E-06 | 0.101521582 | 9 | 72149346 | *GDA* |
| cg19738774 | 0.002105469 | 5.778E-06 | 0.10540129 | 11 | 69011381 | *MRGPRF* |
| cg05836965 | 0.002643348 | 6.01626E-06 | 0.107308843 | 14 | 32933861 | Intergenic |
| cg14633815 | 0.003922013 | 6.46339E-06 | 0.11098727 | 11 | 19713477 | *LOC100126784;NAV2* |
| cg03100191 | -0.002735786 | 6.94147E-06 | 0.111755347 | 17 | 38427727 | *ARHGAP23* |
| cg03525145 | -0.002504796 | 6.96173E-06 | 0.111755347 | 6 | 159725098 | Intergenic |
| cg09016212 | 0.004654927 | 7.20376E-06 | 0.112272431 | 12 | 52006855 | *GRASP* |
| cg08073882 | 0.002448545 | 7.29205E-06 | 0.1125557 | 4 | 6200653 | *JAKMIP1* |
| cg12314713 | 0.002699706 | 7.50852E-06 | 0.113440498 | 17 | 48997213 | *IGF2BP1* |
| cg19247495 | -0.00234544 | 8.11333E-06 | 0.116287343 | 10 | 96561212 | *TM9SF3* |
| cg23632539 | 0.001586273 | 8.98543E-06 | 0.120869134 | 1 | 235644088 | *MIR5096;GNG4* |
| cg15356923 | 0.002761757 | 9.09429E-06 | 0.120869134 | 3 | 68931859 | *FAM19A4* |
| cg02997497 | -0.004818832 | 9.19613E-06 | 0.120869134 | 3 | 68004805 | *FAM19A1* |
| cg22235132 | -0.002012567 | 9.22817E-06 | 0.120869134 | 6 | 53455056 | Intergenic |
| cg14135770 | 0.003300472 | 9.46984E-06 | 0.120869134 | 16 | 90022627 | *GAS8* |
| cg01016102 | -0.002884086 | 9.48712E-06 | 0.120869134 | 7 | 33107195 | *RP9* |
| cg03495011 | -0.003563632 | 1.02203E-05 | 0.12782741 | 20 | 63125822 | Intergenic |
| cg15832313 | -0.002548317 | 1.06247E-05 | 0.131196845 | 16 | 5067384 | *C16orf89* |
| cg17741501 | 0.002613369 | 1.09143E-05 | 0.132772018 | 15 | 28832346 | Intergenic |
| cg03868473 | 0.002348713 | 1.10004E-05 | 0.132772018 | 9 | 137430224 | *NOXA1* |
| cg09106624 | 0.003209828 | 1.11438E-05 | 0.132932636 | 12 | 53938716 | *HOXC13* |
| cg16460004 | -0.002686252 | 1.15088E-05 | 0.134467352 | 4 | 184805943 | *ACSL1* |
| cg13429998 | -0.004388298 | 1.19882E-05 | 0.137264793 | 1 | 41919275 | *HIVEP3* |
| cg16158396 | -0.002721957 | 1.28473E-05 | 0.138586934 | 6 | 109465888 | *MICAL1;ZBTB24* |
| cg20298541 | 0.005737919 | 1.338E-05 | 0.138586934 | 19 | 3721384 | *TJP3* |
| cg07607126 | 0.001985028 | 1.34125E-05 | 0.138586934 | 5 | 159100460 | *EBF1* |
| cg08493160 | -0.002767779 | 1.37324E-05 | 0.138586934 | 2 | 55840367 | Intergenic |
| cg16236766 | 0.00487453 | 1.38418E-05 | 0.138586934 | 2 | 73202202 | *NOTO* |

**Supplementary Table 2: Top 50 Associations from the Factor TN EWAS**

| CpG | Beta | *p*-value | FDR | Chr | Position | Gene |
| --- | --- | --- | --- | --- | --- | --- |
| cg26033520 | -0.414665191 | 1.74896E-10 | 0.000280757 | 10 | 72244312 | ASCCI (near) |
| cg23156469 | -0.193924955 | 5.83624E-08 | 0.031229379 | 1 | 179069287 | *FAM20B* |
| cg15356923 | 0.214977677 | 8.59245E-08 | 0.034483251 | 3 | 68931859 | *FAM19A4* |
| cg16802508 | -0.234560544 | 1.94797E-07 | 0.058489366 | 11 | 67303266 | *SSH3* |
| cg10487211 | -0.145599065 | 2.56327E-07 | 0.058489366 | 6 | 53279548 | *ELOVL5* |
| cg26692003 | 0.298402316 | 2.91485E-07 | 0.058489366 | 3 | 13021664 | *IQSEC1* |
| cg08639389 | -0.228573226 | 3.36913E-07 | 0.060093399 | 4 | 26994266 | *STIM2* |
| cg02086025 | -0.511824634 | 4.04895E-07 | 0.064223794 | 2 | 240156682 | Intergenic |
| cg05141871 | -0.435294175 | 4.40086E-07 | 0.064223794 | 11 | 67304020 | *SSH3* |
| cg14921128 | -0.157685688 | 1.08729E-06 | 0.078547425 | 16 | 28177681 | *XPO6* |
| cg07145988 | -0.16705409 | 1.12492E-06 | 0.078547425 | 1 | 8632252 | *RERE* |
| cg09977701 | -0.181779047 | 1.2168E-06 | 0.078547425 | 3 | 53026702 | *SFMBT1* |
| cg24042452 | -0.167044994 | 1.2444E-06 | 0.078547425 | 1 | 156335872 | *CCT3;C1orf182* |
| cg10089338 | -0.157494363 | 1.28409E-06 | 0.078547425 | 3 | 120072950 | *GSK3B* |
| cg17336761 | -0.148590752 | 1.35219E-06 | 0.078547425 | 1 | 64879588 | *JAK1* |
| cg00814751 | -0.199519633 | 1.37786E-06 | 0.078547425 | 5 | 176645168 | *EIF4E1B* |
| cg19402463 | -0.166898951 | 1.40846E-06 | 0.078547425 | 9 | 130110399 | *GPR107* |
| cg00983583 | 0.237634645 | 1.41899E-06 | 0.078547425 | 6 | 117265991 | *VGLL2* |
| cg16300586 | -0.207028964 | 1.63835E-06 | 0.081317806 | 13 | 54989689 | Intergenic |
| cg20931305 | -0.203858601 | 1.67166E-06 | 0.081317806 | 6 | 16436666 | *ATXN1* |
| cg12120119 | -0.157851638 | 1.79268E-06 | 0.084031172 | 5 | 34719190 | *RAI14* |
| cg18453621 | 0.172902652 | 1.88448E-06 | 0.084031172 | 9 | 126614624 | *LMX1B* |
| cg10047291 | -0.138800183 | 2.10431E-06 | 0.088160165 | 1 | 111441337 | *WDR77* |
| cg05671637 | -0.181742216 | 2.27349E-06 | 0.088160165 | 10 | 94401390 | *TBC1D12* |
| cg12802812 | -0.154813744 | 2.29685E-06 | 0.088160165 | 12 | 120554339 | *RNF10* |
| cg04870523 | 0.223869728 | 2.33466E-06 | 0.088160165 | 3 | 115512590 | Intergenic |
| cg27260459 | -0.17014016 | 2.38591E-06 | 0.088160165 | 4 | 83140479 | Intergenic |
| cg16040306 | -0.263575432 | 2.41401E-06 | 0.088160165 | 1 | 166384639 | Intergenic |
| cg27659440 | 0.200658803 | 2.6151E-06 | 0.090629215 | 17 | 67258128 | Intergenic |
| cg23812637 | -0.139073112 | 2.63359E-06 | 0.090629215 | 1 | 9946481 | *NMNAT1* |
| cg05480110 | 0.277710219 | 2.65347E-06 | 0.090629215 | 3 | 195807982 | *MUC4* |
| cg15631509 | -0.160886083 | 2.92999E-06 | 0.093248119 | 19 | 17159721 | *MYO9B* |
| cg16712866 | -0.143101747 | 2.95858E-06 | 0.093248119 | 16 | 12265439 | *SNX29* |
| cg21454600 | -0.150488442 | 2.99548E-06 | 0.093248119 | 3 | 18730570 | Intergenic |
| cg22021539 | -0.185247866 | 3.07006E-06 | 0.093248119 | 7 | 30279357 | Intergenic |
| cg04661109 | -0.167494296 | 3.18576E-06 | 0.093944309 | 18 | 7803544 | *PTPRM* |
| cg03546163 | -0.27802643 | 3.28058E-06 | 0.093944309 | 6 | 35686585 | *FKBP5* |
| cg19038989 | -0.209561589 | 3.39757E-06 | 0.093944309 | 8 | 140045288 | *TRAPPC9* |
| cg01024069 | -0.203605306 | 3.45016E-06 | 0.093944309 | 14 | 103692540 | *KLC1* |
| cg10892749 | -0.21675551 | 3.52779E-06 | 0.093944309 | 14 | 34324329 | Intergenic |
| cg02674903 | -0.180481338 | 3.60154E-06 | 0.093944309 | 2 | 201187717 | *CASP10* |
| cg04416750 | 0.147002829 | 3.60666E-06 | 0.093944309 | 3 | 98901725 | *DCBLD2* |
| cg11647493 | -0.158489501 | 3.62358E-06 | 0.093944309 | 6 | 4134250 | *PECI* |
| cg05138403 | -0.162035892 | 3.73772E-06 | 0.093944309 | 14 | 93044445 | *ITPK1* |
| cg27270307 | -0.175812281 | 3.8042E-06 | 0.093944309 | 5 | 157385435 | *CYFIP2* |
| cg17272966 | -0.19218463 | 3.81082E-06 | 0.093944309 | 11 | 100405103 | Intergenic |
| cg22799396 | -0.305310363 | 3.86245E-06 | 0.093944309 | 4 | 185472274 | *CCDC110* |
| cg21248060 | 0.298698009 | 3.93738E-06 | 0.094073498 | 7 | 1000320 | *C7orf50* |
| cg04035713 | -0.14888429 | 4.04664E-06 | 0.094144946 | 2 | 197514352 | *MOB4;HSPE1-MOB4* |
| cg06758171 | -0.140656712 | 4.19622E-06 | 0.096230205 | 7 | 131657792 | Intergenic |

**Supplementary Table 3: Full SEM Results**

**KEY TO VARIABLES**

**PTAU181, GFAP, NFL, AB40, AB42**: Raw values of SIMOA markers.

**AGE**: Age at assessment.

**SEX**: coded 1=male, 2=female.

**PTSD**: Current PTSD severity determined by CAPS interview.

**TRAUMA**: Sum of number of lifetime traumatic events endorsed on the Traumatic Life Events Questionnaire.

**APOE4**: # of copies the *APOE* E4 allele (0-2).

**PC1, PC2, PC3**: Genotype determined principle components (used to control for ancestral stratification)

**APOE4xPTSD**: interaction term

**cg26033520, cg23156469, cg15356923, cg130534081**: DNA methylation level of the EWAS significant probes.

**CD8t, CD4t, NK, BCELL, MONO**: DNA methylation estimated cell proportions (used as covariates of the CpG methylation levels).

**SmoS:** DNA methylation-based smoking score.

**STANDARDIZED MODEL RESULTS** (*STDYX Standardization*)

**FACTOR TN LOADINGS**

λ SE p-value

PTAU181 0.415 0.050 0.000

GFAP 0.606 0.050 0.000

NFL 0.677 0.055 0.000

**FACTOR A LOADINGS**

λ SE p-value

AB40 0.945 0.038 0.000

AB42 0.688 0.037 0.000

**FACTOR TN REGRESSED ON**

β SE p-value

AGE 0.581 0.043 0.000

SEX 0.081 0.045 0.071

PTSD -0.140 0.047 0.003

TRAUMA 0.000 0.047 0.995

APOE4 -0.149 0.068 0.028

PC1 0.100 0.043 0.019

PC2 -0.037 0.033 0.260

PC3 0.058 0.030 0.054

APOE4xPTSD 0.155 0.070 0.027

cg26033520 -0.238 0.047 0.000

cg23156469 -0.142 0.047 0.003

cg15356923 0.177 0.055 0.001

cg130534081 -0.025 0.044 0.569

SmoS 0.007 0.046 0.873

**FACTOR A REGRESSED ON**

β SE p-value

AGE 0.330 0.035 0.000

SEX 0.091 0.039 0.021

PTSD 0.027 0.046 0.568

TRAUMA 0.112 0.044 0.010

APOE4 0.024 0.064 0.707

PC1 0.196 0.039 0.000

PC2 -0.065 0.041 0.116

PC3 -0.022 0.041 0.583

APOE4xPTSD -0.043 0.064 0.500

cg130534081 -0.152 0.047 0.001

cg26033520 -0.110 0.037 0.003

cg23156469 -0.057 0.043 0.187

cg15356923 0.105 0.040 0.008

SmoS 0.005 0.140 0.888

**cg26033520 REGRESSED ON**

β SE p-value

AGE 0.008 0.041 0.844

SEX 0.244 0.040 0.000

PTSD -0.036 0.045 0.421

TRAUMA -0.052 0.038 0.172

APOE4 -0.042 0.070 0.545

PC1 0.159 0.045 0.000

PC2 0.020 0.052 0.698

PC3 -0.016 0.051 0.759

APOE4xPTSD 0.040 0.067 0.550

CD8t 0.088 0.041 0.030

CD4t -0.146 0.040 0.000

NK -0.101 0.043 0.020

BCELL 0.015 0.067 0.826

MONO 0.019 0.043 0.664

SmoS -0.005 0.040 0.164

**cg23156469 REGRESSED ON**

β SE p-value

AGE -0.033 0.043 0.448

SEX 0.073 0.041 0.073

PTSD -0.039 0.043 0.365

TRAUMA -0.145 0.062 0.266

APOE4 -0.145 0.062 0.019

PC1 0.077 0.048 0.112

PC2 -0.184 0.047 0.000

PC3 -0.036 0.039 0.355

APOE4xPTSD 0.187 0.066 0.005

CD8t 0.041 0.044 0.359

CD4 t -0.004 0.042 0.921

NK 0.065 0.039 0.097

BCELL -0.181 0.126 0.153

MONO 0.015 0.051 0.767

SmoS -0.037 0.038 0.329

**cg15356923 REGRESSED ON**

β SE p-value

AGE 0.234 0.035 0.000

SEX -0.030 0.042 0.353

PTSD 0.042 0.044 0.341

TRAUMA -0.069 0.048 0.147

APOE4 0.003 0.058 0.959

PC1 -0.022 0.050 0.662

PC2 0.014 0.040 0.719

PC3 0.004 0.026 0.893

APOE4xPTSD -0.079 0.058 0.173

CD8t 0.147 0.054 0.006

CD4t 0.008 0.042 0.854

NK -0.007 0.036 0.845

BCELL 0.191 0.145 0.190

MONO -0.011 0.049 0.831

SmoS -0.013 0.035 0.711

**cg130534081 REGRESSED ON**

β SE p-value

AGE -0.065 0.040 0.105

SEX 0.104 0.038 0.006

PTSD 0.037 0.045 0.411

TRAUMA -0.142 0.041 0.001

APOE4 -0.035 0.060 0.557

PC1 0.016 0.049 0.737

PC2 -0.160 0.049 0.001

PC3 -0.002 0.036 0.963

APOE4xPTSD 0.061 0.062 0.327

CD8t -0.009 0.046 0.841

CD4t -0.023 0.042 0.581

NK 0.066 0.033 0.042

BCELL -0.307 0.134 0.021

MONO 0.040 0.034 0.377

SmoS -0.006 0.034 0.861

**CORRELATIONS**

*r* SE p-value

FACTOR TN WITH

FACTOR A 0.472 0.071 0.000

cg26033520 WITH

cg23156469 0.122 0.049 0.013

cg15356923 0.018 0.060 0.763

cg130534081 0.078 0.055 0.159

cg23156469 WITH

cg15356923 -0.160 0.092 0.081

cg130534081 0.291 0.082 0.000

cg15356923 WITH

cg130534081 -0.211 0.107 0.049

**Supplementary Data Files (EWAS summary statistics) Note.** SE=standard error; P=p-value; FDR=false discovery rate adjusted p-value. Chr=chromosome containing the CpG (hg38). BP=base-pair position of the CpG (hg38). Gene=name of the gene containing the CpG, from UCSC Genome Browser database; hg38=Human genome build 38.
